# Sex Differences in the Association Between Body Composition, Sarcopenic Obesity, and Periodontitis: Mediating Role of Oxidative Stress Markers

**DOI:** 10.3290/j.ohpd.c_2770

**Published:** 2026-08-12

**Authors:** Yike Yao, Jiahui Sun, Yanting Zou, Yuan Zhang, Yuanyuan Jiang, Jun Ji

**Affiliations:** a Yike Yao Dentist, Nanjing Stomatological Hospital, Affiliated Hospital of Medical School, Institute of Stomatology, Nanjing University, Nanjing, China. Conceptualization, methodology, investigation, wrote original draft, commented on previous versions of the manuscript, read and approved the final manuscript.; b Jiahui Sun Dentist, Nanjing Stomatological Hospital, Affiliated Hospital of Medical School, Institute of Stomatology, Nanjing University, Nanjing, China. Formal analysis, validation, data curation, commented on previous versions of the manuscript, read and approved the final manuscript.; c Yanting Zou Dentist, Nanjing Stomatological Hospital, Affiliated Hospital of Medical School, Institute of Stomatology, Nanjing University, Nanjing, China. Software, visualization, investigation, commented on previous versions of the manuscript, read and approved the final manuscript.; d Yuan Zhang Dentist, Nanjing Stomatological Hospital, Affiliated Hospital of Medical School, Institute of Stomatology, Nanjing University, Nanjing, China. Validation, reviewed and edited the manuscript, commented on previous versions of the manuscript, read and approved the final manuscript.; e Yuanyuan Jiang Dentist, Nanjing Stomatological Hospital, Affiliated Hospital of Medical School, Institute of Stomatology, Nanjing University, Nanjing, China. Validation, reviewed and edited the manuscript, commented on previous versions of the manuscript, read and approved the final manuscript.; f Jun Ji Professor, Nanjing Stomatological Hospital, Affiliated Hospital of Medical School, Institute of Stomatology, Nanjing University, Nanjing, China. Supervision, funding acquisition, project administration, reviewed and edited manuscript, commented on previous versions of the manuscript, read and approved the final manuscript.

**Keywords:** muscle-to-fat ratio, NHANES, oral health, periodontitis, sarcopenic obesity, sex differences.

## Abstract

**Purpose:**

This study aims to examine, in a sex-stratified manner, the associations of fat mass index (FMI), muscle mass index (MMI), and the muscle-to-fat ratio (MFR) with oral health as represented by periodontitis (PD), and to quantify the mediating role of systemic inflammatory and oxidative stress (OS) markers in these associations.

**Materials and Methods:**

This research drew on cross-sectional data from the National Health and Nutrition Examination Survey (NHANES 2011-2014; n=3,553). Confounder-adjusted weighted logistic regression models determined sex-stratified associations. Restricted cubic spline (RCS) regression evaluated non-linear MFR-PD relationships. Mediation analysis quantified the contribution of OS markers.

**Results:**

Among women, lower MFR was associated with a statistically significantly increased PD risk (OR=0.49, 95% CI:0.34-0.72, p=0.001). Higher FMI (OR=1.59, 95% CI:1.15-2.19, p=0.008) and MMI (OR=1.86, 95% CI:1.13-3.07, p=0.018) also conferred increased risk. No statistically significant associations were observed in men. Serum albumin mediated 15.1% (p= 0.040) of the association between MFR and PD, 27.9% (p= 0.040) of the association between MMI and PD, and 23.4% (p<0.001) of the association between FMI and PD. Subgroup analyses revealed no statistically significant interaction effects (all p> 0.05).

**Conclusion:**

In women, a lower MFR is independently associated with higher PD prevalence, while higher FMI and MMI are also associated with elevated PD prevalence. Serum albumin partly explains these associations, suggesting that oxidative stress may be one of the pathways linking body composition imbalance to compromised oral health. No comparable associations were observed in men. For clinicians, MFR together with serum albumin may serve as accessible and low-cost markers for identifying women, particularly those in the peri-menopausal period, who could benefit from early periodontal screening and targeted nutritional or lifestyle interventions. Prospective cohort studies and interventional trials are warranted to confirm causality and to evaluate whether improving body composition translates into measurable benefits for oral health.

Periodontitis (PD) is a chronic inflammatory disease characterized by the progressive destruction of the periodontal supporting tissues (encompassing the gums, alveolar bone, and periodontal ligaments),^24^ and represents a major contributor to compromised oral health worldwide. PD is the predominant cause of tooth loss in adults (accounting for 45% to 50% of cases) and is bidirectionally associated with systemic diseases such as diabetes and cardiovascular diseases.^18^ Recent NHANES-based evidence further indicates that edentulism is independently associated with higher all-cause mortality, underscoring that compromised oral health carries prognostic implications well beyond the oral cavity.^15^ The Global Burden of Disease Study (GBD) reports that PD ranks as the sixth most prevalent disease worldwide. PD imposes significant physical and psychological burden on patients and leads to substantial productivity losses, and has therefore become a major global public health challenge.^13,28^ A recent bibliometric review has further confirmed its broad associations with nutritional, metabolic, cardiovascular, and musculoskeletal disorders, highlighting growing interest in the connection between oral and systemic health.^21^

The pathogenesis of PD is fundamentally a dynamic imbalance caused by the interaction between the immune system of the host and the dysbiotic microbial community in the dental plaque biofilm. Specific pathogenic bacteria in the biofilm (such as *Porphyromonas gingivalis* and *Aggregatibacter actinomycetemcomitans*) activate the TLR signaling pathway of the host, leading to the excessive release of pro-inflammatory cytokines (e.g., IL-6 and TNF-α), which ultimately result in bone resorption and loss of periodontal attachment.^29^ Beyond local tissue damage, these inflammatory mediators can enter the bloodstream and induce systemic low-grade inflammation, thereby affecting overall health.^6^ A network of inflammatory and oxidative stress (OS) mediators is thought to link the oral environment with the systemic circulation. Serum cytokines have been recognized as key signals connecting periodontal disease activity to systemic inflammatory burden, and their circulating profiles can serve as informative readouts of both periodontal and overall health status.^1^ In parallel, the field is gradually moving beyond purely clinical phenotyping, including probing depth and attachment loss, toward host response biomarkers derived from saliva, blood, and gingival crevicular fluid, which provide more biologically grounded indicators of oral disease processes.^8^ At the molecular level, the balance between reactive oxygen species, reactive nitrogen species, and antioxidant defences critically shapes oral health. Under homeostatic conditions, nitric oxide generated from oral microbial nitrate reduction supports microbial eubiosis and immune regulation, whereas chronic inflammation shifts this balance toward nitric oxide scavenging, NOS uncoupling, and oxidative tissue damage.^31^ Together, these observations suggest that circulating inflammatory and OS markers may not only mirror periodontal status but also reflect a broader continuum of oral health that closely interacts with systemic metabolism.

Among the systemic conditions that may interact with oral health through inflammatory and oxidative pathways, sarcopenic obesity (SO) has received increasing attention. SO is a comorbid condition characterized by a decrease in skeletal muscle mass index (MMI) and an abnormal increase in body fat percentage.^11,12^ The prevalence of SO increases with age.^30^ It is estimated that approximately 11% of older adults globally suffer from SO, with a sharp rise after the age of 70 years.^16^ SO is associated with an increased the risk of metabolic syndrome, functional impairment, and mortality. The pathophysiology of SO is driven by the interplay of four main factors: chronic inflammation, insulin resistance, hormonal disturbances, and oxidative stress (OS),^5,34^ among which OS plays a central role. OS damages muscle homeostasis through three primary mechanisms: mitochondrial dysfunction, endoplasmic reticulum stress, and disrupted regulation of muscle mass.^20^

Notably, SO and PD share OS as a core pathophysiological hub, and the two conditions may promote each other through a bidirectional cycle. In patients with SO, excessive secretion of pro-inflammatory cytokines (e.g., IL-6 and TNF-α) from visceral fat tissue activates neutrophil NADPH oxidase, resulting in a significant increase in serum reactive oxygen species (ROS) levels. This damages periodontal tissue cells, worsens periodontal inflammation, and promotes bone resorption.^2^ Conversely, PD serves as a local chronic infection site, from which inflammatory mediators and OS markers such as 8-hydroxy-2’-deoxyguanosine (8-OHdG) in gingival crevicular fluid enter systemic circulation.^4,9^ These mediators bind to proteins such as serum albumin to form advanced glycation end-products (AGEs). AGEs have been shown to inhibit key signaling pathways in skeletal muscle cells, impair muscle protein synthesis, and promote catabolism.^22^ In addition, studies have shown that periodontal pathogens (e.g., *Porphyromonas gingivalis*) and their secreted proteases may trigger systemic inflammatory responses or directly affect liver function, thereby lowering serum albumin levels and significantly weakening the systemic antioxidant capacity of the body.^4,17^ Taken together, systemically elevated OS induced by SO may compromise periodontal barrier function and accelerate bone resorption. Meanwhile, locally propagated inflammation from PD, together with increased systemic OS markers such as glycated albumin, may further disrupt muscular metabolic homeostasis. This bidirectional interaction ultimately forms a self-reinforcing pathological cycle between SO and PD.^35^

Despite this conceptual progress, direct evidence linking body composition imbalance to oral health remains limited. Previous research on the association between obesity and PD has mostly relied on single metrics such as body mass index (BMI),^26^ and has not fully considered the distinct pathophysiological impact of SO as a “high-oxidation, high-inflammation” phenotype. In addition, the role of sex-specific mechanisms^6^ linking body composition to oral health remains insufficiently quantified, and the role of circulating inflammatory and OS markers as potential mediators has not been systematically examined. The nationally representative NHANES dataset has long supported epidemiological studies of periodontitis, including the development of self-reported risk-prediction algorithms.^14^ Building on this resource, the present study integrates dual-energy X-ray absorptiometry (DXA) body composition data, periodontal clinical indicators, and serum OS markers. The aim of this study was to examine the sex-specific associations of fat mass index (FMI), muscle mass index (MMI), and the muscle-to-fat ratio (MFR) with oral health as represented by PD, and to quantify the mediating role of inflammatory and OS markers, particularly serum albumin. We hypothesized that an unfavorable body composition (higher FMI and lower MMI/MFR) is associated with poorer oral health, particularly in women, and that this association is partly mediated by systemic inflammatory and OS markers, especially serum albumin.

## MATERIALS AND METHODS

### Ethics Statement

Ethics review and approval were waived for this study, as it was conducted using publicly available, de-identified data from the National Health and Nutrition Examination Survey (NHANES). Because NHANES data are anonymized and collected with informed consent by the National Center for Health Statistics, additional ethical approval was not required for this secondary data analysis.

### Data Source and Study Population

This research used data from NHANES 2011-2014. NHANES is a nationally representative cross-sectional survey assessing the health and nutritional indicators among US-Americans. It employs a stratified multistage probability sampling design to accurately represent the civilian, non-institutionalized US population.

**Fig 1:**
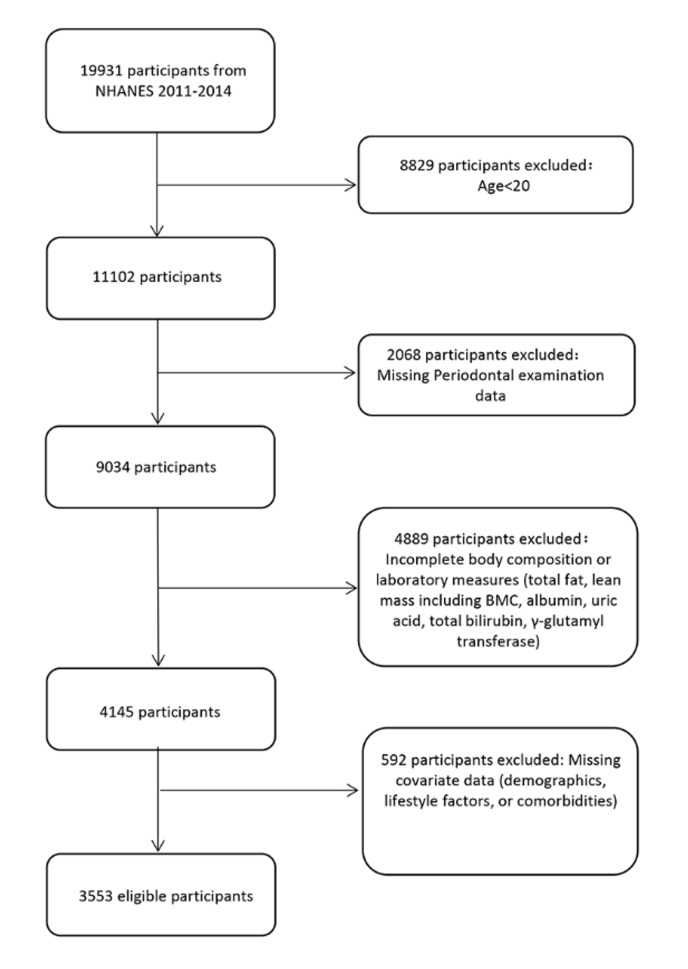
Flowchart of study population selection (based on NHANES 2011-2014).

Among the 19,931 participants in the NHANES 2011-2014, minors were excluded, leaving 11,102 adults aged ≥ 20 years. We then excluded 2068 participants with missing periodontal examination data and 4889 participants lacking data on total fat, lean mass (excluding BMC), albumin, uric acid, total bilirubin, and γ-glutamyltransferase. Subsequently, 4145 eligible participants remained. Additionally, 592 participants were excluded due to missing other demographic, lifestyle, or comorbidity data. Ultimately, 3553 participants were included (Fig 1).

The NHANES protocol was approved by the Institutional Review Board of the Centers for Disease Control and Prevention (CDC) at the National Center for Health Statistics (NCHS) (Approval Number: Protocol #2011-17). Written informed consent was obtained from all participants or their legal guardians. As this was a secondary analysis of publicly available de-identified data, no additional ethical review was required by our institution.

### Diagnostic Criteria for PD

The periodontal examination data from NHANES are stored in the standardized document OHXPER.DAT, containing measurements of clinical attachment loss (CAL) and probing depth for all oral sites. Certified examiners collected data using a Williams probe, covering six sites: mesial buccal, buccal central, distal buccal, mesial lingual, lingual central, and distal lingual.

PD was classified based on the case definitions of the 2012 CDC/American Academy of Periodontology (AAP), including mild PD, moderate PD, and severe PD. Severe PD was defined as ≥ 2 interproximal sites with CAL ≥ 6mm (non-adjacent teeth) and ≥ 1 interproximal site with probing depth ≥ 5mm. Moderate PD was defined as ≥ 2 interproximal sites with CAL ≥ 4mm (non-adjacent teeth) or ≥ 2 interproximal sites with probing depth ≥ 5mm (non-adjacent teeth). Mild PD was defined as ≥ 2 interproximal sites with CAL ≥ 3mm and ≥ 2 interproximal sites with probing depth ≥4mm (non-adjacent teeth), or ≥ 1 site with probing depth ≥ 5mm.^27^ In this study, we consolidated mild, moderate, and severe PD into a unified PD group. Participants not meeting the diagnostic criteria were assigned to the non-PD group.

### Anthropometric Measurements and Whole-Body DXA Scans

Height and weight were measured by trained health staff using standardized protocols. Following the standard operating procedures established by the International Society for Clinical Densitometry (ISCD), whole-body DXA scans were performed by trained technicians using a Hologic fan-beam densitometer (Hologic; Bedford, MA USA) to assess body composition indicators. Data from the DXA scans included total fat mass (TFM), bone mineral content (BMC), and total lean mass (TLM) values.^25^

The following indices were calculated: fat mass index (FMI, kg/m^2^) = TFM / height^2^; MMI (kg/m^2^) = (TLM – BMC) / height^2^; muscle-to-fat ratio (MFR) = (TLM – BMC) / TFM.

### Covariates

Based on prior evidence, covariates included demographic information, lifestyle, and health conditions. Demographic information consisted of age, sex (male or female), race (Mexican American, other Hispanic, non-Hispanic White, non-Hispanic Black, non-Hispanic Asian, and other races including multiracial), marital status (MS; married, previously married, or never married), educational level (<high school, high school, or above), and family income-to-poverty ratio (PIR; < 1 or ≥ 1). Lifestyle included smoking status (SS) and alcohol consumption (ALC). SS was determined from the question: Have you ever smoked at least 100 cigarettes? Participants reporting yes were classified as smokers. Participants answering no were classified as non-smokers. ALC was determined from the question: Have you ever consumed at least 12 alcoholic drinks in any one year? Participants reporting yes were classified as drinkers. Participants answering no were classified as non-drinkers. Health conditions included hypertension, high cholesterol, and diabetes, which were determined from the question: Have doctors or health professionals ever told you that you have any of the following conditions? Participants reporting yes were classified as having the condition. Participants answering no were classified as not having the condition.

### Statistical Analysis

This study employed a complex multistage sampling design. Given the differential selection probabilities for different samples, inverse probability weighting was applied to ensure the accuracy of the results and population representativeness. All statistical analyses accounted for the complex survey design by incorporating the sample weights, stratification variables (SDMVSTRA), and primary sampling unit (PSU) indicators (SDMVPSU) provided by NHANES. The mobile examination center (MEC) examination weights (WTMEC2YR) were used for analyses involving variables collected at the MEC, as recommended by the NCHS. Since data from two NHANES cycles (2011–2012 and 2013–2014) were combined, the 4-year sample weights were calculated as one-half of WTMEC2YR, following NCHS guidelines for combining survey cycles.

Baseline features of the study population were presented using descriptive statistics. Normally distributed continuous variables were expressed as mean ± standard deviation. Intergroup comparisons were performed using independent samples t-tests. Non-normally distributed continuous variables were presented as median (interquartile range) [M (Q1, Q3)]. Intergroup comparisons were performed using the Kruskal-Wallis H-test. Categorical variables were presented as frequencies (percentages) [n (%)]. Intergroup comparisons were performed using the chi-squared (X^2^) test or Fisher’s exact test.

A weighted logistic regression (WLR) model was used to examine the association between body composition indicators (FMI, MMI, MFR) and PD, with adjustment for confounders. Restricted cubic spline (RCS) regression was employed to examine the potential non-linear relationship between the MFR indicator and PD. Subgroup analysis by age, PIR, SS, ALC, diabetes, hypertension, and hyperlipidemia was performed to examine the association between body composition and PD, the robustness of the association between exposure and outcome across various subgroups, and the interaction among variables. Multicollinearity among covariates was assessed using variance inflation factors (VIF), with all values below 5 indicating no substantial collinearity. FMI, MMI and MFR were modeled in separate equations to avoid structural collinearity. The linearity of continuous exposures was examined using restricted cubic splines, and effect modification in subgroup analyses was tested using likelihood ratio tests.

Mediation analysis was performed to estimate the mediating effect of OS markers (albumin, uric acid, total bilirubin, γ-glutamyltransferase) on the association between body composition indicators (FMI, MMI, MFR) and PD.

Two sensitivity analyses were further performed in the female population to assess the robustness of the main findings. First, to address potential bias arising from categorical grouping of periodontitis severity, survey-weighted ordinal logistic regression was conducted, modeling periodontitis as a four-level ordered outcome (none < mild < moderate < severe) under the same three sequentially adjusted models. Second, to account for additional potential confounding by lifestyle and inflammatory factors, an extended model (Model 4) was constructed by further adjusting Model 3 for physical activity and white blood cell (WBC) count. Physical activity was derived from the Physical Activity Questionnaire using metabolic equivalent (MET) scores, with participants reaching ≥600 MET-min/week classified as physically active in line with WHO recommendations; otherwise, they were classified as inactive. WBC count was obtained from the Complete Blood Count laboratory file and used as a marker of systemic inflammation.

Data analysis was performed using R software (version 4.3.0) and RStudio (version 2025.05.0+496). Two-tailed p<0.05 signified statistical significance.

## RESULTS

The data presented in this study are openly available in the National Health and Nutrition Examination Survey (NHANES) database (https://www.cdc.gov/nchs/nhanes/index.html).

### Baseline Features of the Participants

In total, 3553 participants were included, of whom 1752 were female and 1801 were male.

The baseline features of 1752 female participants from NHANES 2011-2014 are provided in Table 1. The median age of the female population was 45 years, and the prevalence of PD was 29.1%. The median age of individuals with PD (48 years) was statistically significantly higher than that of those without PD (45 years) (p < 0.001). Statistically significant differences were detected in socioeconomic characteristics. The proportion of individuals with less than a high-school education in the PD group (20%) was statistically significantly higher than that in the non-PD group (10%). The proportion of participants with PIR < 1 in the PD group (20%) was statistically significantly higher than that in the non-PD group (13%). The proportion of non-Hispanic Black individuals in the PD group (17%) was statistically significantly higher than that in the non-PD group (9.6%) (all p < 0.001). Behavioral risk factors analysis showed that the current smoking rate in the PD group (56%) was statistically significantly higher than that in the non-PD group (38%) (p < 0.001). The prevalence of hypertension in the PD group (34%) was statistically significantly higher than that in the non-PD group (26%) (p= 0.047). The prevalence of diabetes in the PD group (9.6%) tended to be higher than that in the non-PD group (6.9%) (p= 0.077). Statistically significant differences in body composition indicators were observed. FMI in the PD group (1.21) was higher than the non-PD group (1.09) (p < 0.001). MMI in the PD group (1.67) was higher than the non-PD group (1.60) (p < 0.001). MFR in the PD group (1.39) was lower than in the non-PD group (1.47) (p < 0.001). Statistically significant differences in OS markers were detected. Serum albumin in the PD group (4.1 g/dl) was lower than in the non-PD group (4.2 g/dl) (p < 0.001). Total bilirubin in the PD group (0.5 mg/dl) was lower than in the non-PD group (0.6 mg/dl) (p=0.046). γ-glutamyltransferase in the PD group (17 U/L) was higher than in the non-PD group (15 U/L; p=0.002). The baseline features of 1801 male participants from NHANES 2011-2014 are provided in Table 2. The median age of the male population was 45 years, and the prevalence of PD was 43.8%. The median age of individuals with PD (46 years) was statistically significantly higher than those without PD (43 years) (p < 0.001). Statistically significant differences were detected in socioeconomic characteristics. The proportion of individuals with less than a high-school education in the PD group (23%) was statistically significantly higher than that in the non-PD group (8.6%). The proportion of participants with PIR < 1 in the PD group (20%) was statistically significantly higher than that in the non-PD group (8.3%). The proportion of Mexican American individuals in the PD group (13%) was statistically significantly higher than the non-PD group (6.9%). The proportion of non-Hispanic Black individuals in the PD group (12%) was statistically significantly higher than the non-PD group (7.3%) (all p<0.001). The MS analysis showed that the proportion of previously married individuals in the PD group (19%) was statistically significantly higher than the non-PD group (11%) (p<0.001). Behavioral risk factors analysis showed that the current smoking rate in the PD group (59%) was statistically significantly higher than that in the non-PD group (41%) (p<0.001). The prevalence of hyperlipidemia in the PD group (33%) was statistically significantly lower than that in the non-PD group (39%) (p=0.044). The prevalence of diabetes in the PD group (9.7%) tended to be higher than that in the non-PD group (6.5%) (p=0.068). The drinking rate (92%) tended to be higher in the PD group than that in the non-PD group (88%) (p=0.098). Statistically significant differences in OS markers were noted. Serum albumin in the PD group (4.30 g/dl) was lower than in the non-PD group (4.40 g/dl) (p<0.001). Uric acid in the PD group (5.90 mg/dl) was lower than in the non-PD group (6.00 mg/dl; p=0.028).Total bilirubin in the PD group (0.70 mg/dl) was lower than in the non-PD group (0.70 mg/dl; p<0.001).

**Table 1:** Baseline features of the female population (stratified by periodontitis status, NHANES 2011-2014)

Characteristic	Overall	No periodontitis	Periodontitis	p-value^1^
	N = 1752	N = 1243	N = 509	
Age, y (IQR)	45 (38, 52)	45 (37, 51)	48 (41, 53)	<0.001
**Race (%)**				<0.001
Mexican American	213 (8.2%)	132 (6.9%)	81 (12%)	
Other Hispanic	169 (5.8%)	122 (5.5%)	47 (6.8%)	
Non-Hispanic White	718 (67%)	556 (71%)	162 (55%)	
Non-Hispanic Black	380 (11%)	236 (9.6%)	144 (17%)	
Non-Hispanic Asian	213 (4.8%)	151 (4.5%)	62 (5.8%)	
Other race, including multi-racial	59 (2.6%)	46 (2.6%)	13 (2.8%)	
**Educational attainment (%)**				<0.001
Above high school	1131 (70%)	871 (74%)	260 (56%)	
Below high school	284 (12%)	164 (10%)	120 (20%)	
High school	337 (18%)	208 (16%)	129 (25%)	
**Marital status (%)**				<0.001
Formerly married	397 (21%)	258 (18%)	139 (29%)	
Married	1097 (68%)	815 (72%)	282 (54%)	
Never married	258 (12%)	170 (10%)	88 (17%)	
**PIR (%)**				<0.001
<1	372 (15%)	224 (13%)	148 (20%)	
>=1	1380 (85%)	1019 (87%)	361 (80%)	
**Smoking (%)**				<0.001
Yes	651 (42%)	418 (38%)	233 (56%)	
No	1101 (48%)	825 (62%)	276 (44%)	
**Alcohol consumption (%)**				0.136
Yes	1176 (77%)	864 (78%)	312 (74%)	
No	576 (23%)	379 (22%)	197 (26%)	
**Diabetes (%)**				0.077
Yes	168 (7.6%)	101 (6.9%)	67 (9.6%)	
No	1584 (92.4%)	1142 (93.1%)	442 (90.4%)	
**Hypertension (%)**				0.047
Yes	517 (28%)	343 (26%)	174 (34%)	
No	1235 (72%)	900 (74%)	335 (66%)	
**Hyperlipidemia (%)**				0.406
Yes	514 (31%)	358 (30%)	156 (33%)	
No	1238 (69%)	885 (70%)	353 (67%)	
Serum albumin, g/dl (IQR)	4.20 (4.00, 4.40)	4.20 (4.00, 4.40)	4.10 (4.00, 4.30)	<0.001
Uric acid, mg/dl (IQR)	4.60 (3.80, 5.30)	4.50 (3.80, 5.30)	4.60 (4.00, 5.50)	0.059
Total bilirubin, mg/dl (IQR)	0.60 (0.50, 0.70)	0.60 (0.50, 0.70)	0.50 (0.40, 0.70)	0.046
γ-glutamyltransferase, U/L (IQR)	16 (11, 24)	15 (11, 23)	17 (13, 26)	0.002
FMI (IQR)	1.13 (0.86, 1.41)	1.09 (0.83, 1.40)	1.21 (1.00, 1.49)	<0.001
MMI (IQR)	1.61 (1.45, 1.84)	1.60 (1.44, 1.81)	1.67 (1.50, 1.92)	<0.001
MFR (IQR)	1.45 (1.23, 1.72)	1.47 (1.25, 1.78)	1.39 (1.21, 1.58)	<0.001
^1^ Design-based Kruskal-Wallis test; Pearson’s X^2^: Rao & Scott adjustment. IQR: interquartile range.				

**Table 2:** Baseline features of the male population (stratified by periodontitis status, NHANES 2011-2014)

Characteristic	Overall	No periodontitis	Periodontitis	p-value^1^
	N = 1801	N = 1012	N = 789	
Age, y (IQR)	45 (37, 52)	43 (36, 51)	46 (40, 54)	<0.001
**Race (%)**				<0.001
Mexican American	231 (9.3%)	102 (6.9%)	129 (13%)	
Other Hispanic	146 (5.6%)	81 (5.2%)	65 (6.4%)	
Non-Hispanic White	778 (68%)	482 (73%)	296 (60%)	
Non-Hispanic Black	340 (9.1%)	167 (7.3%)	173 (12%)	
Non-Hispanic Asian	241 (5.0%)	143 (4.9%)	98 (5.1%)	
Other race, including multi-racial	65 (3.2%)	37 (3.0%)	28 (3.5%)	
**Educational attainment (%)**				<0.001
Above high school	1028 (62%)	684 (72%)	344 (46%)	
Below high school	345 (14%)	135 (8.6%)	210 (23%)	
High school	428 (24%)	193 (19%)	235 (31%)	
**Marital status (%)**				<0.001
Formerly married	260 (14%)	118 (11%)	142 (19%)	
Married	1,264 (71%)	731 (75%)	533 (66%)	
Never married	277 (15%)	163 (14%)	114 (15%)	
**PIR (%)**				<0.001
<1	358 (13%)	146 (8.3%)	212 (20%)	
≥1	1443 (87%)	866 (92%)	577 (80%)	
**Smoking (%)**				<0.001
Yes	910 (48%)	439 (41%)	471 (59%)	
No	891 (52%)	573 (59%)	318 (41%)	
**Alcohol consumption (%)**				0.098
Yes	1,548 (89%)	852 (88%)	696 (92%)	
No	253 (11%)	160 (12%)	93 (8%)	
**Diabetes (%)**				0.068
Yes	159 (7.7%)	75 (6.5%)	84 (9.7%)	
No	1642 (92.3%)	937 (93.5%)	705 (90.3%)	
**Hypertension (%)**				0.148
Yes	526 (29%)	283 (28%)	243 (32%)	
No	1275 (71%)	729 (72%)	546 (68%)	
**Hyperlipidemia (%)**				0.044
Yes	615 (37%)	378 (39%)	237 (33%)	
No	1186 (63%)	634 (61%)	552 (67%)	
Serum albumin, g/dl (IQR)	4.40 (4.20, 4.60)	4.40 (4.20, 4.60)	4.30 (4.20, 4.50)	<0.001
Uric acid, mg/dl (IQR)	6.00 (5.20, 6.80)	6.00 (5.30, 6.80)	5.90 (5.10, 6.80)	0.028
Total bilirubin, mg/dl (IQR)	0.70 (0.60, 0.90)	0.70 (0.60, 0.90)	0.70 (0.50, 0.80)	<0.001
γ-glutamyltransferase, U/L (IQR)	23 (17, 37)	23 (17, 34)	25 (17, 41)	0.101
FMI (IQR)	0.79 (0.64, 0.99)	0.78 (0.64, 0.97)	0.80 (0.64, 1.01)	0.445
MMI (IQR)	1.97(1.81, 2.16)	1.95(1.82, 2.15)	1.99(1.81, 2.18)	0.178
MFR (IQR)	2.47(2.10, 2.95)	2.46(2.11, 2.97)	2.47(2.09, 2.94)	0.831
^1^ Design-based Kruskal-Wallis test; Pearson’s X^2^: Rao & Scott adjustment. IQR: interquartile range.				

### Association Between Body Composition and PD Prevalence

WLR was employed to evaluate the association between body composition indices (FMI, MFR, MMI) and PD separately in male and female populations, while adjusting for confounding factors. Model 1 was unadjusted. Model 2 adjusted for age, race, education level, MS, and PIR. Model 3 additionally adjusted for lifestyle factors(SS, ALC) and clinical comorbidities (hypertension, hyperlipidemia, and diabetes).

For the female population (Table 3), in Model 1 (OR = 0.457; 95% CI: 0.333, 0.627; p<0.001), Model 2 (OR=0.555; 95% CI: 0.392, 0.786; p=0.002), and Model 3 (OR = 0.493; 95% CI: 0.339, 0.718; p=0.001), continuous MFR was statistically significantly inversely associated with PD prevalence. Furthermore, MFR was categorized into tertiles; the lowest tertile (T1) was designated as the reference group across all the models. In Model 1 (T3: OR = 0.561; 95% CI: 0.396, 0.795; p= 0.002), Model 2 (T3: OR = 0.624; 95% CI: 0.473, 0.943; p= 0.026), and Model 3 (T3: OR = 0.593; 95% CI: 0.403, 0.873; p= 0.012), participants with higher MFR levels (T2, T3) had a statistically significantly lower PD prevalence relative to those with lower MFR levels (T1).

**Table 3:** Weighted logistic regression analysis of body composition (MFR, FMI, MMI) and periodontitis risk in females

Variables	Model 1		Model 2		Model 3	
	OR (95%CI)	p-value	OR (95%CI)	p-value	OR (95%CI)	p-value
MFR continuous	0.457 (0.333, 0.627)	<0.001	0.555 (0.392, 0.786)	0.002	0.493 (0.339, 0.718)	0.001
**MFR tertile**						
T1	-		-		-	
T2	0.956 (0.677, 1.350)	0.793	1.014 (0.693, 1.485)	0.939	1.004 (0.698, 1.445)	0.981
T3	0.561 (0.396, 0.795)	0.002	0.624 (0.437, 0.943)	0.026	0.593 (0.403, 0.873)	0.012
MMI continuous	2.046 (1.390, 3.012)	<0.001	1.737 (1.149, 2.627)	0.011	1.863 (1.130, 3.073)	0.018
**MMI tertile**						
T1	-		-		-	
T2	1.314 (0.890, 1.940)	0.162	1.301 (0.854, 1.983)	0.207	1.294 (0.806, 2.077 )	0.262
T3	1.844 (1.345, 2.529)	<0.001	1.606 (1.128, 2.286)	0.011	1.680 (1.081, 2.609)	0.024
FMI continuous	1.718 (1.315, 2.244)	<0.001	1.453 (1.090, 1.937)	0.013	1.586 (1.149, 2.190)	0.008
**FMI tertile**						
T1	-		-		-	
T2	2.263 (1.656, 3.093 )	<0.001	2.075 (1.487, 2.896)	<0.001	2.218 (1.521, 3.234)	<0.001
T3	2.209 (1.574, 3.102)	<0.001	1.838 (1.269, 2.661)	0.003	2.037 (1.365, 3.041)	0.002
Model 1: unadjusted; Model 2: adjusted for age, race, educational attainment, marital status, PIR; Model 3: adjusted for age, race, educational attainment, marital status, PIR, smoking, alcohol consumption, diabetes, hypertension, hyperlipidemia.						

In Model 1 (OR = 1.718; 95% CI: 1.315, 2.244; p<0.001), Model 2 (OR = 1.453; 95% CI: 1.090, 1.937; p= 0.013), and Model 3 (OR = 1.586; 95% CI: 1.149, 2.190; p=0.008), continuous FMI was significantly positively associated with PD prevalence. Additionally, we categorized FMI into tertiles and designated the lowest tertile (T1) as the reference group across all the models. In Model 1 (T2: OR = 2.263; 95% CI: 1.656, 3.093; p<0.001; T3: OR = 2.209; 95% CI: 1.574, 3.102; p<0.001), Model 2 (T2: OR = 2.075; 95% CI: 1.487, 2.896; p<0.001; T3: OR = 1.838; 95% CI: 1.269, 2.661; p=0.003), and Model 3 (T2: OR = 2.218; 95% CI: 1.521, 3.234; p<0.001; T3: OR = 2.037; 95% CI: 1.365, 3.041; p=0.002), participants with higher FMI levels (T2, T3) had a significantly higher PD prevalence than those with lower FMI levels (T1).

In Model 1 (OR = 2.046; 95% CI: 1.390, 3.012; p<0.001), Model 2 (OR = 1.737; 95% CI: 1.149, 2.627; p=0.011), and Model 3 (OR = 1.863; 95% CI: 1.130, 3.073; p=0.018), continuous MMI was statistically significantly positively associated with PD prevalence. In addition, we categorized MMI into tertiles and designated the lowest tertile (T1) as the reference group across all the models. In Model 1 (T3: OR = 1.844; 95% CI: 1.345, 2.529; p<0.001), Model 2 (T3: OR = 1.606; 95% CI: 1.128, 2.286; p=0.011), and Model 3 (T3: OR = 1.680; 95% CI: 1.081, 2.609; p=0.024), participants with higher MMI levels (T2, T3) had a statistically significantly higher prevalence of PD than those with lower MMI levels (T1).

For the male population, in all three models, MFR, MMI, and FMI were not statistically significantly associated with PD (Table 4).

**Table 4:** Weighted logistic regression analysis of body composition (MFR, FMI, MMI) and periodontitis risk in males

Variables	Model 1		Model 2		Model 3	
	OR (95%CI)	p-value	OR (95%CI)	p-value	OR (95%CI)	p-value
MFR continuous	1.033 (0.873, 1.222)	0.697	1.030 (0.843, 1.257)	0.763	1.011 (0.832, 1.228)	0.909
**MFR tertile**						
T1	-		-		-	
T2	0.978 (0.711, 1.345)	0.885	0.896 (0.618, 1.301)	0.546	0.883 (0.608, 1.281)	0.484
T3	0.899 (0.629, 1.286)	0.549	0.905 (0.600, 1.367)	0.62	0.870 (0.581, 1.301)	0.469
MMI continuous	1.351 (0.862, 2.119 )	0.182	1.473 (0.945, 2.295)	0.084	1.567 (0.987, 2.488)	0.056
**MMI tertile**						
T1	-		-		-	
T2	0.834 (0.567, 1.225 )	0.342	0.840 (0.562, 1.256)	0.377	0.861 (0.560, 1.325)	0.469
T3	1.113 (0.842, 1.472)	0.44	1.196 (0.908, 1.575)	0.189	1.240 (0.914, 1.684)	0.153
FMI continuous	1.133 (0.726, 1.769)	0.57	1.135 (0.711, 1.811)	0.579	1.179 (0.761, 1.827)	0.435
**FMI tertile**						
T1	-		-		-	
T2	0.848 (0.648, 1.111 )	0.223	0.816 (0.589, 1.130)	0.207	0.837 (0.583, 1.203)	0.311
T3	1.140 (0.794, 1.639)	0.465	1.131 (0.761, 1.681)	0.522	1.180 (0.798, 1.745)	0.381
Model 1: unadjusted; Model 2: adjusted for age, race, educational attainment, marital status, PIR; Model 3: adjusted for age, race, educational attainment, marital status, PIR, smoking, alcohol consumption, diabetes, hypertension, hyperlipidemia.						

### RCS Regression Analysis

**Fig 2:**
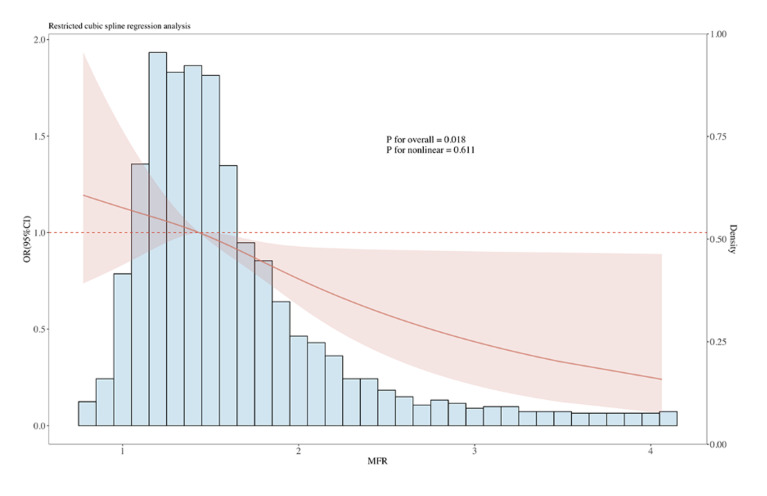
Restricted cubic spline plot of the association between muscle-to-fat ratio (MFR) and periodontitis prevalence in females.

The RCS regression model was employed to examine the nonlinear relationship between MFR and the risk of PD in the female population (Fig 2). After extensively adjusting for covariates, encompassing age, race, educational level, MS, household PIR, SS, ALC, hypertension, diabetes, and hypercholesterolemia, the results showed a statistically significant overall association between MFR and the risk of PD (p overall = 0.018). However, the nonlinear trend was not significant (p nonlinear = 0.611). These results suggested that there was an approximately linear negative association between MFR and the risk of PD. As MFR levels diminished, the risk of PD continuously increased. No apparent threshold effect or plateau phase was detected.

### Subgroup Analysis

**Fig 3:**
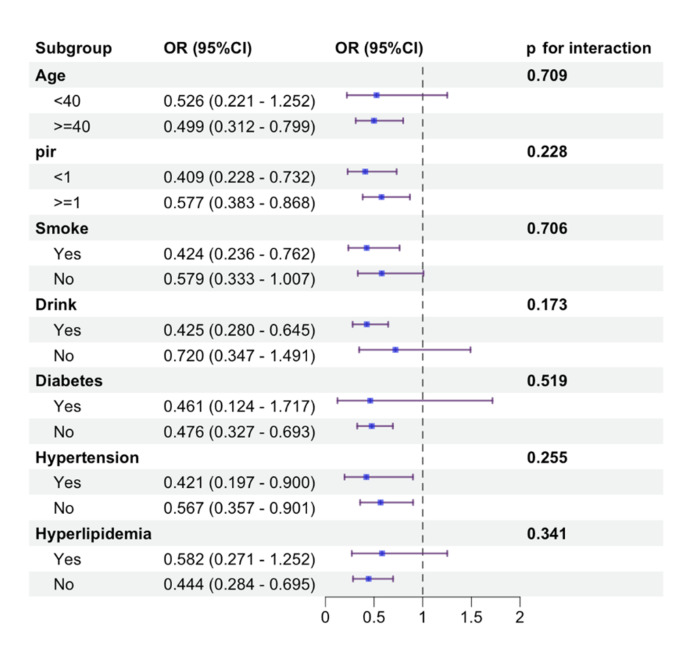
Subgroup analysis and interaction test (forest plot) of the association between muscle-to-fat ratio (MFR) and periodontitis prevalence in females.

The WLR results revealed a statistically significant positive association between body composition and PD prevalence in females. Further subgroup analysis was performed for the female group. The results of the subgroup analysis are provided in Fig 3. Though the strength of the association between MFR and PD varied across different subgroups, no statistically significant interaction was detected (all p for interaction > 0.05). Specifically, the association between higher MFR and statistically significantly lower risks of PD was detected in the subgroups of age ≥ 40 years, higher household income (PIR ≥ 1), smokers, drinkers, and those without diabetes, hypertension, or hyperlipidemia. This association was not statistically significant in subgroups of age < 40 years, non-smokers, non-drinkers, and those with diabetes or hyperlipidemia.

### Mediation Analysis

To clarify the role of oxidative stress in linking body composition to periodontitis, mediation analysis was conducted in the female population to break down the association of MFR, MMI, and FMI with PD prevalence into a direct effect (the residual association not transmitted through the mediator) and an indirect effect (the association transmitted through the candidate OS mediator). Four OS biomarkers were evaluated separately: serum albumin (LBXSAL), uric acid (LBXSUA), total bilirubin (LBXSTB), and γ-glutamyltransferase (LBXSGTSI). Bootstrap resampling (1000 replicates) was used to derive 95% confidence intervals (CIs), and the proportion mediated was calculated as the ratio of the indirect effect to the total effect. All models were adjusted for the covariates included in Model 3.

**Fig 4a to c:**
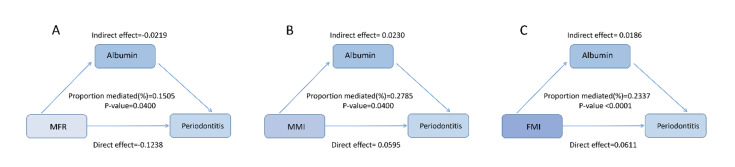
Path diagrams illustrating the mediation role of serum albumin (LBXSAL) in the associations between body composition indices (MFR, FMI, MMI) and periodontitis in females.

Among the four candidate mediators, serum albumin was the only OS biomarker showing a statistically significant indirect effect across all three body composition indices, whereas uric acid, total bilirubin, and γ-glutamyltransferase did not exhibit statistically significant mediation (Table 5, Fig 4).

**Table 5:** Mediation analysis of oxidative stress markers in the association between body composition (MFR, FMI, MMI) and periodontitis prevalence in females

Indicators	OS markers	Indirect effect (95%CI)	Direct effect (95%CI)	Proportion mediated (95%CI)	p-value
MFR	Serum albumin	-0.0219 (-0.0385,-0.0001)	-0.1238 (-0.2130,-0.0600)	0.1505 (0.0058,0.2800)	0.0400*
	Uric acid	-0.0045 (-0.0219,0.0100)	-0.1432 (-0.2329,-0.0800)	0.0306 (-0.0840,0.1800)	0.8000
	Total bilirubin	-0.0058 (-0.0133,-0.0000)	-0.1419 (-0.2291,-0.0700)	0.0391 (-0.0167,0.1100)	0.1400
	γ-glutamyltransferase	-0.0002 (-0.0047, 0.0000)	-0.1479 (-0.2360, -0.0800)	0.0017 (-0.0248, 0.0300)	0.8600
MMI	Serum albumin	0.0230 (0.0005,0.0400)	0.0595 (0.0242,0.0900)	0.2785 (0.0081,0.5800)	0.0400*
	Uric acid	0.0041 (-0.0141,0.0200)	0.0781 (0.0421,0.1000)	0.0503 (-0.1896,0.3200)	0.8200
	Total bilirubin	0.0058 (-0.0027,0.0100)	0.0766 (0.0450,0.1000)	0.0699 (-0.0329,0.2300)	0.1800
	γ-glutamyltransferase	0.0002 (-0.0022, 0.0000)	0.0820 (0.0553,0.1000)	0.0031 (-0.0267, 0.0500)	0.8600
FMI	Serum albumin	0.0186 (0.0035,0.0400)	0.0611 (0.0161,0.0900)	0.2337 (0.0465,0.6300)	<0.0001*
	Uric acid	0.0024 (-0.0118,0.0200)	0.0772 (0.0400,0.1100)	0.0302 (-0.1894,0.2800)	0.9600
	Total bilirubin	0.0046 (-0.0023,0.0100)	0.0751 (0.0430,0.1000)	0.0581 (-0.0297,0.1700)	0.2000
	γ-glutamyltransferase	0.0002 (-0.0028, 0.0000)	0.0794 (0.0479, 0.1100)	0.0024 (-0.0299, 0.0500)	0.8800
Indirect effect: the portion of the association between body composition and periodontitis transmitted through the candidate OS mediator. Direct effect: the residual association between body composition and periodontitis after accounting for the mediator. Proportion mediated: the ratio of the indirect effect to the total effect, reflecting the magnitude of mediation. All models were adjusted for age, race/ethnicity, educational attainment, marital status, poverty-to-income ratio, smoking status, alcohol consumption, diabetes, hypertension, and hyperlipidemia. Confidence intervals were derived from 1000 bootstrap resamples. * p < 0.05 for the indirect effect.					

Specifically, the indirect effect through albumin was −0.0219 (95% CI: −0.0385 to −0.0001) for MFR, 0.0230 (95% CI: 0.0005 to 0.0400) for MMI, and 0.0186 (95% CI: 0.0035 to 0.0400) for FMI. The corresponding direct effects remained statistically significant: −0.1238 (95% CI: −0.2130 to −0.0600) for MFR, 0.0595 (95% CI: 0.0242 to 0.0900) for MMI, and 0.0611 (95% CI: 0.0161 to 0.0900) for FMI. Albumin accounted for 15.05% (95% CI: 0.58% to 28.00%; p=0.040) of the association between MFR and PD, 27.85% (95% CI: 0.81% to 58.00%; p=0.040) of the association between MMI and PD, and 23.37% (95% CI: 4.65% to 63.00%; p<0.001) of the association between FMI and PD.

The direction of the indirect and direct effects was concordant for each exposure: both pathways were inverse for MFR and both were positive for MMI and FMI, indicating that albumin transmits, rather than offsets, part of the association between body composition and PD. Because the direct effects remained statistically significant after accounting for albumin, these findings represent partial mediation, suggesting that serum albumin contributes to – but does not fully explain – the link between body composition and periodontitis in females.

In contrast, the 95% CIs of the indirect effects through uric acid, total bilirubin, and γ-glutamyltransferase all crossed the null, and the corresponding p-values were non-significant (all p>0.05), indicating that these biomarkers did not transmit a detectable portion of the association between body composition and PD in this cohort.

### Sensitivity Analyses

To address potential bias from grouping periodontitis severity categories, ordinal logistic regression was performed in women, treating periodontitis as an ordered four-level outcome (none < mild < moderate < severe). Across all three sequentially adjusted models, lower MFR and higher MMI and FMI were each statistically significantly associated with greater odds of more severe periodontitis, with consistent monotonic trends across tertiles (fully adjusted: MFR per 1-unit increase, OR = 0.506, 95% CI 0.357–0.716, p<0.001; MMI per 1-unit increase, OR = 1.773, 95% CI 1.120–2.805, p=0.014; FMI T3 vs T1, OR = 2.401, 95% CI 1.571–3.670, p<0.001), confirming that the associations are robust to the categorization scheme of periodontitis severity (Table 6).

**Table 6:** Sensitivity analysis using weighted ordinal logistic regression of body composition (MFR, FMI, MMI) and periodontitis severity in females (NHANES 2011–2014)

Variables	Model 1		Model 2		Model 3	
	OR (95%CI)	p-value	OR (95%CI)	p-value	OR (95%CI)	p-value
MFR (per 1 unit)	0.461 (0.340-0.624)	<0.001	0.562 (0.405-0.779)	<0.001	0.506 (0.357-0.716)	<0.001
MFR T2 vs T1	0.649 (0.473-0.891)	0.007	0.714 (0.520-0.981)	0.038	0.680 (0.494-0.935)	0.017
MFR T3 vs T1	0.456 (0.270-0.768)	0.003	0.591 (0.318-1.100)	0.097	0.501 (0.260-0.965)	0.039
MMI (per 1 unit)	1.979 (1.369-2.862)	<0.001	1.693 (1.144-2.503)	0.008	1.773 (1.120-2.805)	0.014
MMI T2 vs T1	1.554 (1.188-2.034)	0.001	1.329 (0.988-1.787)	0.06	1.362 (0.973-1.907)	0.072
MMI T3 vs T1	1.648 (1.208-2.248)	0.002	1.467 (1.056-2.037)	0.022	1.557 (1.083-2.240)	0.017
FMI (per 1 unit)	1.692 (1.316-2.175)	<0.001	1.431 (1.096-1.869)	0.008	1.536 (1.146-2.059)	0.004
FMI T2 vs T1	1.818 (1.141-2.897)	0.012	1.487 (0.890-2.483)	0.13	1.542 (0.934-2.547)	0.091
FMI T3 vs T1	2.717 (2.028-3.640)	<0.001	2.204 (1.546-3.141)	<0.001	2.401 (1.571-3.670)	<0.001
Model 1: unadjusted; Model 2: adjusted for age, race, educational attainment, marital status, PIR; Model 3: adjusted for age, race, educational attainment, marital status, PIR, smoking, alcohol consumption, diabetes, hypertension, hyperlipidemia.						

In a further sensitivity analysis additionally adjusting for physical activity and WBC count, the associations remained materially unchanged: lower MFR (continuous OR = 0.491, 95% CI 0.329–0.734, p=0.002; T3 vs T1 OR = 0.595, 95% CI 0.394–0.897, p=0.017), higher MMI (continuous OR = 1.820, 95% CI 1.038–3.193, p=0.038; T3 vs T1 OR = 1.655, 95% CI 1.024–2.676, p=0.041), and higher FMI (continuous OR = 1.573, 95% CI 1.099–2.253, p=0.017; T3 vs T1 OR = 2.041, 95% CI 1.340–3.108, p=0.003) all remained statistically significantly associated with periodontitis (Table 7), indicating that the observed associations are independent of physical activity level and systemic inflammation.

**Table 7:** Sensitivity analysis of body composition (MFR, FMI, MMI) and periodontitis prevalence in females with additional adjustment for physical activity and white blood cell count (Model 4, NHANES 2011–2014)

Variables	OR (95%CI)	p-value
**Continuous**		
MFR (per 1 unit)	0.491 (0.329-0.734)	0.002
MMI (per 1 unit)	1.820 (1.038-3.193)	0.038
FMI (per 1 unit)	1.573 (1.099-2.253)	0.017
**Tertiles**		
MFR T2 vs T1	1.007 (0.697-1.453)	0.97
MFR T3 vs T1	0.595 (0.394-0.897)	0.017
MMI T2 vs T1	1.284 (0.777-2.122)	0.3
MMI T3 vs T1	1.655 (1.024-2.676)	0.041
FMI T2 vs T1	2.224 (1.510-3.277)	<0.001
FMI T3 vs T1	2.041 (1.340-3.108)	0.003
Model 4: adjusted for age, race, educational attainment, marital status, PIR, smoking, alcohol consumption, diabetes, hypertension, hyperlipidemia, physical activity, WBC.		

## DISCUSSION

This study provides new evidence of the sex-specific association between SO and PD risk through sex-stratified analysis. In women, lower MFR was statistically significantly associated with higher PD prevalence, while higher FMI and MMI were also positively correlated with the risk of PD. The RCS regression analysis further confirmed an approximately linear dose-response relationship between MFR and the risk of PD in women, indicating that as MFR decreased, the risk of PD increased continuously without a clear threshold effect. In contrast, no statistically significant association was detected between body composition indices and PD prevalence in men, suggesting that the underlying mechanism may differ by sex. Among the OS biomarkers, serum albumin was identified as a key mediator of the association between body composition and PD, statistically significantly mediating the associations of MFR, MMI, and FMI with PD. The inverse association between MFR and PD was observed in subgroups of age ≥ 40 years, higher household income (PIR ≥ 1), smokers, drinkers, and those without diabetes, hypertension, or hyperlipidemia. Nevertheless, no statistically significant interaction was observed for any variable.

The sex-specific pattern observed here invites a reconsideration of how previous studies have framed the obesity-periodontitis link. Past studies on the associations between obesity and PD have several limitations. Firstly, they rely heavily on single indicators such as BMI or waist circumference^26^ and overlook the pathological significance of SO, a “high oxidation-high inflammation” phenotype. Furthermore, sex is treated only as a confounder, not as a potential effect modifier. Moreover, evidence that OS mediates the link between body composition imbalance and periodontal destruction is lacking.^3,23,26^ The present study advances this field in three respects. First, by jointly assessing FMI, MMI, and MFR, we establish MFR as a key protective factor for PD in women. Second, sex-stratified analysis reveals that the SO-related periodontal risks are statistically significant only in women. Third, serum albumin is identified as a core mediator of OS, with its statistically significant mediating effect offering preliminary mechanistic clues to the muscle loss–OS dysregulation–periodontal destruction pathway.

A finding that may appear counterintuitive is the positive, rather than protective, association between MMI and PD in women. However, in this population, higher MMI was closely accompanied by higher FMI, indicating that women with greater muscle mass also carried more fat. Under these conditions, the inflammatory and oxidative burden from excess adiposity likely outweighs any protective benefit of muscle mass alone. This interpretation is supported by the finding that MFR, which captures the relative balance between muscle and fat rather than absolute quantity, showed the most consistent inverse association with periodontitis. It suggests that metabolic health depends more on body composition balance than on muscle mass in isolation.

This pattern of susceptibility in females and resistance in males is underpinned by complex biological mechanisms. Susceptibility in females can be attributed to hormonal regulatory networks, differences in fat distribution, and sexual dimorphism in albumin function. Estrogen may inhibit macrophage polarization to the M1 type by upregulating adiponectin, and potentially reduce IL-6 release from adipose tissue while activating SOD via the Nrf2/ARE pathway,^19^ potentially reducing TNF-α levels in gingival crevicular fluid. The sharp post-menopausal decline in estrogen secretion reduces myokines such as irisin, weakening their promotion of osteogenic differentiation in periodontal ligament stem cells.^7^ Additionally, visceral fat in females has been reported to accumulate pro-inflammatory adipokines (IL-6, leptin). When MFR decreases, adipose expansion may activate the NF-κB pathway, possibly amplifying oxidative damage in the gingiva. Furthermore, estrogen receptor α (ERα) directly regulates the expression of hepatic albumin genes (ALB). Under low MFR conditions, females may experience a statistically significantly greater decline in albumin than males, which could weaken free radical scavenging capacity and exacerbate oxidative damage. Although males might present with an SO phenotype, no statistically significant association with PD risk was detected in our analysis. This may stem from the bidirectional effects of androgens. Testosterone maintains muscle mass by inhibiting myostatin but simultaneously upregulates the expression of NADPH oxidase subunit NOX4, promoting the production of ROS. The combined effects of pro-oxidation and anti-oxidation may achieve a balance.^32^ Additionally, among males, their mitochondrial antioxidant capacity is greater, enabling them to buffer the stress of adipose expansion. Although these mechanisms together provide a biologically plausible framework for the sex-specific pattern observed, they remain largely inferential in the context of a cross-sectional study, and the present findings should be interpreted as biologically plausible associations rather than definitive evidence of causal mechanisms.

Beyond its role as an antioxidant marker, serum albumin reflects multiple interrelated physiological processes, with its antioxidant capacity representing one of several dimensions of its biological role, alongside systemic inflammation and nutritional status. In the context of sarcopenic obesity, these dimensions are tightly interconnected: visceral adiposity-driven inflammation enhances ROS production while concurrently suppressing hepatic albumin synthesis, and impaired nutritional status further depletes antioxidant substrates such as cysteine. These processes converge on the redox imbalance pathway, supporting the role of albumin as an oxidative stress marker in our mediation analysis while recognizing that its inflammatory and nutritional dimensions act in parallel. Accordingly, the partial mediation by albumin observed here is best interpreted as an integrative inflammatory–nutritional–oxidative signal rather than a marker of oxidative stress in isolation, which also suggests that the systemic consequences of sarcopenic obesity may extend beyond periodontitis to a broader range of oral health outcomes.

It is important to situate these findings within a broader oral health framework rather than treating periodontitis as an isolated outcome. Periodontitis represents the clinically measurable endpoint of a continuum of inflammatory oral disease. The systemic inflammatory and oxidative burden associated with sarcopenic obesity, reflected in part by reduced serum albumin, is likely to affect oral health more broadly, including subclinical gingival inflammation, mucosal immune defence, and alveolar bone homeostasis. Circulating pro-inflammatory cytokines such as IL-6 and TNF-α, which are chronically elevated in sarcopenic obesity, have been shown to impair periodontal tissue repair and promote bone resorption even below the diagnostic threshold for periodontitis. Our results therefore suggest that body composition imbalance may compromise oral health along the entire inflammatory spectrum, with periodontitis serving as the available clinical proxy in NHANES. Future studies should incorporate a wider range of oral health outcomes, including gingivitis indices, tooth loss, and salivary inflammatory markers, to more fully characterize this relationship. From a translational perspective, the present findings carry several practical implications. Conceptually, they shift attention from the isolated effect of fat mass to the dynamic balance between muscle and adipose tissue, positioning MFR as a more informative composition marker than BMI for PD risk stratification. The mediating role of albumin further indicates that metabolic oxidative stress may act as an amplifier of periodontal damage, and combining MFR with serum albumin could provide a low-cost screening approach for identifying peri-menopausal women at elevated risk, supporting the development of sex-specific preventive strategies.^14^

Based on the nationally representative NHANES sample, this research is pioneering in revealing sex-specific associations and the mediating role of OS. However, several limitations should be acknowledged. (i) The cross-sectional design is a fundamental limitation that precludes causal inference. While we observed associations between body composition indices (MFR, FMI, MMI) and periodontitis, we cannot determine the temporal sequence or directionality of these relationships. Reverse causation remains a plausible alternative explanation, as severe periodontitis may lead to mastication difficulties and subsequent malnutrition, altering body composition. Therefore, the associations reported in this study should be interpreted as correlational rather than causal, and prospective cohort studies with repeated measurements are essential to establish temporal precedence and confirm the direction of these relationships. (ii) Behavioral exposures, including smoking and alcohol consumption, were self-reported and subject to recall bias, and residual confounding from unmeasured lifestyle factors cannot be fully excluded. (iii) Data on oral hygiene practices, dietary patterns (e.g., protein intake, Healthy Eating Index), and classic inflammatory markers such as high-sensitivity C-reactive protein were unavailable or substantially incomplete in the analytic sample; their absence limits a more granular evaluation of lifestyle–phenotype interactions and of inflammation-specific pathways, although our sensitivity analysis additionally adjusting for physical activity and white blood cell count showed materially unchanged associations. (iv) Serum albumin, while a well-established integrative marker of systemic redox balance, is jointly influenced by nutritional status, inflammation, and hepatic synthesis; its role here should therefore be interpreted as one dimension of a broader inflammatory–nutritional signal, and future studies incorporating more specific oxidative stress indicators (e.g., 8-OHdG, malondialdehyde, SOD/CAT/GPx) are warranted to disentangle these pathways. (v) Internationally standardized diagnostic thresholds for sarcopenic obesity—particularly for MFR—are currently lacking, as existing consensus criteria (e.g., FNIH^33^; EWGSOP2^10^) focus primarily on muscle mass or physical performance rather than muscle-to-fat balance.^10,33^ MMI, FMI, and MFR were therefore modeled mainly as continuous variables, with tertile categorization used only for relative risk stratification within this U.S.-based sample; the findings should be interpreted as relative associations rather than generalizable diagnostic cut-offs, and validation in other populations is warranted.

## CONCLUSION

This research reveals a sex-specific association between SO and PD. In the female population, decreased MFR is associated with higher PD prevalence, whereas elevated FMI and MMI levels are also positively associated with higher PD prevalence. In the male population, no statistically significant association was detected. As an inflammatory and OS biomarker, serum albumin partially mediates the association between body composition (MFR, FMI, MMI) and PD, suggesting a potential pathway through which muscle-fat metabolic imbalance may relate to broader periodontal and oral health. However, due to the cross-sectional nature of this study, causal relationships and temporal sequences cannot be determined. Therefore, in oral health risk assessment and prevention strategies for PD, attention should be paid to the MFR status of women, particularly peri-menopausal women. Serum albumin should be considered as a potential biomarker for monitoring systemic inflammatory burden and related oral health risks. Prospective cohort studies with repeated measurements, Mendelian randomization analyses, and interventional trials are essential to confirm causality, establish temporal precedence, and evaluate whether interventions targeting body composition or albumin status can effectively reduce periodontitis risk and improve overall oral health.

## ACKNOWLEDGEMENTS

This work was supported by the Key Project supported by Medical Science and technology development Foundation, Nanjing Department of Health (ZKX24058); the High-Level Hospital Construction Project of Nanjing Stomatological Hospital, Affiliated Hospital of Medical School, Institute of Stomatology, Nanjing University (0224C013); and the“2015”Cultivation Program for Reserve Talents for Academic Leaders of Nanjing Stomatological School, Medical School of Nanjing Univeristy (0223A209).
